# Bridging traditions: a template analysis of Buddhist and Western psychological approaches to mindfulness

**DOI:** 10.3389/fpsyg.2026.1711922

**Published:** 2026-06-10

**Authors:** Andrew Boxer, Frances Shawyer, Ian Coghlan, Graham Meadows

**Affiliations:** Monash University, Melbourne, VIC, Australia

**Keywords:** Buddhist psychology, MBCT, MiCBT, mindfulness, mindfulness-based interventions, Satipaṭṭhāna Sutta, second-generation MBIs, template analysis

## Abstract

**Objectives:**

Over recent decades, Western psychology has drawn extensively from Buddhism to create effective mindfulness-based interventions (MBIs). To identify opportunities for deeper integration, this study compared the Satipaṭṭhāna Sutta, a foundational Buddhist text on mindfulness, with two established MBIs for mental health.

**Methods:**

Template analysis was used to systematically compare the Satipaṭṭhāna Sutta with Mindfulness-based Cognitive Therapy (MBCT) and Mindfulness-integrated Cognitive Behavior Therapy (MiCBT) using Anālayo’s breakdown of the Sutta into 15 subcategories as a framework. Similarity between MBIs and the traditional text was assessed using a seven-point Likert scale, with inter-rater reliability employed to examine validity and reliability.

**Results:**

Findings indicate that MBCT and MiCBT align more closely with the first two foundations of mindfulness - awareness of the body and feelings. However, as the Sutta progresses into feeling tones, cognition, and ethics, the alignment diminishes. MiCBT demonstrated slightly greater similarity with Buddhist principles than MBCT (3.4 versus 2.7) but both MBIs largely omit states of mind and the philosophical and liberative dimensions central to Buddhist mindfulness.

**Discussion:**

Feeling tone was identified as a key area for development: improving the differentiation between sensory experiences and interpretations by incorporating Buddhist cognition (e.g., mental states and factors) offers a nuanced two-stage framework for emotion regulation. The omission of mindfulness of mind and dhammas in both MBIs risks neglecting the ethical and wisdom-based elements central to Buddhist practice, potentially narrowing their transformative scope. Future research might explore how these adaptations could be applied in secular therapeutic contexts.

## Introduction

Over recent decades, Western psychology has increasingly drawn on concepts and practices from Buddhist traditions, particularly mindfulness (Krägeloh, 2013). This has led to the development of many effective psychological interventions, most notably mindfulness-based interventions (MBIs). While mindfulness occupies a central role in Buddhist teachings as part of a comprehensive ethical and soteriological path aimed at liberation from suffering (Anālayo, 2003; Thanissaro and DeGraff, 2012), Western psychological applications typically conceptualize mindfulness as present-moment, non-judgmental awareness to improve mental health outcomes such as stress reduction, emotion regulation and resilience (Shapiro et al., 2006; Thanissaro and DeGraff, 2012; Zhang et al., 2021).

These interventions commonly integrate psychological techniques like cognitive-behavioral and, less commonly, psychodynamic, approaches to help individuals observe thoughts and emotions with greater awareness and less reactivity (Kelly, 2025; Krägeloh, 2013). Although this approach has significantly increased the accessibility of mindfulness within clinical contexts, critics argue that it has resulted in a streamlined and secularized version that downplays core ethical, philosophical, and spiritual dimensions central to traditional Buddhist practice (Sedlmeier, 2023).

Gethin (2011) identifies four perspectives on the development of contemporary MBIs: (1) as a distortion of Buddhist aims, (2) as an accessible vehicle for Buddhist teachings, (3) as a culturally adapted yet principled modernization, and (4) as an innovative fusion of Western psychology and Buddhist techniques. A central critique aligned with the first perspective is that MBIs often misunderstand or narrow the concept of mindfulness itself (Anālayo, 2003). In traditional Buddhist contexts, mindfulness (*sati*) refers not merely to present-moment awareness but to an active process of remembering and aligning one’s thoughts and actions with the ethical and moral principles central to Buddhism (Anālayo, 2003). This traditional approach to mindfulness was designed to help practitioners cultivate qualities like compassion, wisdom, and self-awareness while correcting unwholesome mental states and behaviours (Anālayo, 2003; Dreyfus, 2011; Purser and Milillo, 2015). Rather than only observing thoughts and sensations, practitioners were encouraged to recognize negative mental states, apply antidotes, and foster positive qualities, aiming for ethical transformation in line with the broader Buddhist goal of reducing suffering. By comparison, contemporary adaptations of mindfulness, which focus more on stress reduction and emotional regulation, often downplay the ethical and evaluative aspects that are central to Buddhist practice (Dreyfus, 2011). This divergence highlights a key difference between secular mindfulness and its traditional Buddhist roots.

This paper will explore these themes further by analyzing the differences and similarities between traditional Buddhist mindfulness, based on the Satipaṭṭhāna Sutta, and two contrasting MBIs that specifically target mental health, namely Mindfulness-Based Cognitive Therapy (MBCT; Segal et al., 2002; Segal et al., 2013) and Mindfulness-Integrated Cognitive Behavior Therapy (MiCBT; Cayoun et al., 2019). Understanding the differences and harmonies may provide deeper insights into the strengths and limitations of modern MBI’s and help bridge the gap between psychology, spirituality and mental health by highlighting additional Buddhist perspectives that could be integrated into modern applications (Husgafvel, 2016; Shonin et al., 2015).

### Traditional and contemporary conceptualizations of mindfulness

#### Traditional conceptualization

The divergence in conceptualizations of mindfulness reflects differing historical, cultural, and philosophical contexts (Purser and Milillo, 2015). Despite the varied interpretations of mindfulness, it is argued that the Buddha taught only one form of mindfulness (Van Gordon et al., 2015). Mindfulness was first introduced by the Buddha in his first sermon, the Dhammacakkappavattana Sutta, as part of the Noble Eightfold Path, a core teaching and a code of ethical conduct that is crucial for leading a moral life and the cessation of suffering. Further and more elaborate instructions were then given and are described within the Satipaṭṭhāna Sutta or ‘Discourse on the Foundation of Mindfulness’.

Additionally, mindfulness is one facet of how Buddhism conceptualizes consciousness. In the *Lorig*, a foundational Buddhist text that describes knowing and consciousness, mindfulness is just one of 51 aspects or ‘mental factors’ responsible for consciousness (Anālayo, 2003). According to Buddhist psychology (i.e., the classical Buddhist understanding of the mind), the mind is a continuous, moment-to-moment process rather than a fixed or immutable entity. They believe the mind is composed of a primary awareness that perceives objects and accompanying mental factors that shape interpretation, emotion, and reaction (Feldmann, 2016; Gyel-tsen, 2003). These mental factors arise in response to stimuli and influence perception, cognition, and behavior. Since the mind is seen as conditioned by past experiences, Buddhist practice emphasizes cultivating wholesome mental factors while reducing unwholesome ones to progress toward clarity, wisdom, and ultimately liberation.

Mindfulness literally translates to ‘remembering’ and is a quality of the mind that’s responsible for helping one remember or not forget. Historically, early translators (e.g., Rhys-Davids in the 1880s) rendered the Pāli term *sati* as ‘mindfulness,’ while later scholars have emphasized its connotation of *memory* or *recollection* (Gethin, 2011). Generally, what is to be remembered, in this context, is the Buddhist teachings and how to live a virtuous life (Purser and Milillo, 2015). This recollection guides practitioners to assess their thoughts, words, and actions, continually discerning whether they are wholesome or unwholesome. In this sense, mindfulness is not a passive observation but an active, ethical practice (Anālayo, 2003; Thanissaro and DeGraff, 2012), with emerging research also examining spiritual dimensions within contemporary mindfulness-based interventions (e.g., Carmody et al., 2008; Goldberg et al., 2022).

For example, if a practitioner becomes aware of greed, hatred, or other unwholesome or non-virtuous states arising in their mind, they should remember the Buddhist teachings and apply appropriate antidotes. This may include cultivating generosity for greed, loving-kindness for hatred, or wisdom for delusion. Additionally, mindfulness’ role is to foster insight, in conjunction with the Buddhist teachings into everyday experiences, especially regarding impermanence, suffering, and the non-self-nature of existence.

In sum, through mindfulness, observation, and concentrated effort one can use mindfulness as a tool to investigate and observe the present moment, gaining the wisdom necessary to progress toward liberation. Therefore, traditional mindfulness is not merely attentional control or emotional regulation, but viewed as an essential part of a broader, morally integrated spiritual path aimed at enlightenment (Thanissaro and DeGraff, 2012).

#### Contemporary conceptualization

The Western emphasis on mindfulness in meditation is relatively recent compared to its longstanding role in Buddhist traditions. Although Buddhism spans roughly 2,500 years, mindfulness gained broader prominence only in the early twentieth century through Burmese reform movements that made the practice more accessible to lay audiences (Sharf, 2013). This shifted attention away from traditional Samatha (concentration) practices, aimed at cultivating deep meditative absorption, toward “bare awareness” and present moment experience. Scholars view this as characteristic of Buddhist modernism and it has become a transcultural phenomenon, aligning with the third (MBIs as modern adaptations) and fourth perspective (MBIs as innovative fusions) outlined earlier (McMahan, 2008; Monteiro, 2015; Sharf, 2015).

In addition to these historical developments, several formal operational definitions of mindfulness have been proposed in Western psychological literature (e.g., Bishop et al., 2004; Chems-Maarif et al., 2025). These definitions conceptualize mindfulness primarily as a form of attention regulation and present-moment awareness, a framing that differs from traditional Buddhist understandings centered on ethical intention and remembrance (Lustig et al., 2024; Nilsson and Kazemi, 2016; Schmidt, 2011).

In the West, mindfulness is commonly practiced as non-judgmental awareness of thoughts, emotions, and sensations (Kabat-Zinn, 2015; Van Gordon et al., 2015) fostering detachment and insight into the impermanent and unsatisfactory nature of experiences. Critics argue that this “therapeutic turn” can trivialize the ethical and philosophical depth of traditional Buddhist practice (Sharf, 2013). In response, some scholars advocate integrating the deeper dimensions of mindfulness into contemporary MBIs (Sedlmeier et al., 2023). Figures such as Jon Kabat-Zinn (2011), Mark Williams (Williams et al., 2022), Paul Gilbert (2020) and Bruno Cayoun (Cayoun et al., 2019) have highlighted the potential benefits of incorporating broader Buddhist wisdom into contemporary MBIs. For example, Gilbert’s emphasizes integration of compassion and morality in psychology interventions (Gilbert, 2020).

### Traditional and contemporary applications of mindfulness

#### Traditional applications–the Satipaṭṭhāna Sutta

The Satipaṭṭhāna Sutta is a central text for traditional mindfulness practice, offering structured guidance attributed to the Buddha (Anālayo, 2003; Monteiro, 2015; Sedlmeier et al., 2023; Thanissaro and DeGraff, 2012). It outlines four foundations of mindfulness, the body, feelings, mind, and phenomena, which progress from basic to more advanced practices (Anālayo, 2003; Sedlmeier et al., 2023; Thanissaro and DeGraff, 2012). Each foundation builds on the previous one, but they do not need to be practiced in a strictly sequential manner. The practice begins with *mindfulness of body*, particularly through mindfulness of breathing, which helps cultivate concentration and awareness. The practice is advanced through *mindfulness of feeling*, observing feelings as transient and labelling them as pleasant, unpleasant, or neutral. As the practice deepens, the focus shifts to *mindfulness of mind*, and themes of consciousness, observing thoughts and mental formations, and whether one’s conceptions are helpful or not. The final foundation concerns *mindfulness of phenomena or Dhamma,* the broader Buddhist philosophy and instruction for liberation from suffering. By following the progression in the Satipaṭṭhāna Sutta, practitioners move from basic mindfulness practices to insight into the nature of reality, ultimately leading to enlightenment.

Interpretations of the Satipaṭṭhāna Sutta vary, depending on the teacher’s background (Anālayo, 2003; Thanissaro and DeGraff, 2012). Some approaches emphasize a Dhamma-based perspective, integrating Buddhist philosophy into each foundation. Others, however, adopt a ‘no-Dhamma’ approach focused on direct observation without doctrinal framing (Anālayo, 2003; Gale, 2021). These variations allow beginners to develop basic mindfulness skills while offering more philosophically oriented options for advanced practitioners.

#### Contemporary applications

Although many mindfulness programs draw on principles from the Satipaṭṭhāna Sutta, its full depth is rarely apparent in clinical settings (Krägeloh, 2013). Accessible interpretations, such as those by Thich Nhat Hanh, often highlight only the first two foundations; body and feelings (Krägeloh, 2013). However, the broader teachings remain important for understanding challenges that arise in practice.

Consistent with the view of MBIs as an innovative fusion, Western psychology has adopted a pragmatic understanding of mindfulness that incorporates many other Western psychological techniques. For example, contemporary MBIs commonly integrate cognitive and behavioral methods, and explain change via mechanisms such as attentional control and meta-cognitive awareness (Krägeloh, 2013; MacDonald and Olsen, 2020; Sipe and Eisendrath, 2012). Additionally, cognitive-behavioral techniques, including de-centering and de-automatization, can be integrated into the process. These methods help clients develop a heightened self-awareness and reflective capacity, enabling them to observe their thoughts and emotions without immediate judgement or reaction (Krägeloh, 2013; Shapiro et al., 2006; Zhang et al., 2021).

#### First-generation Western integrations: MBCT

MBCT integrates mindfulness with cognitive behavioral therapy (CBT) techniques to target recurrent depression in recovered patients (Segal et al., 2002, 2013).

It was adapted from Mindfulness-Based Stress Reduction (MBSR), which, like MBCT, is classified as a first-generation mindfulness-based intervention within the present framework (Kabat-Zinn, 1982, 2003; Van Gordon and Shonin, 2020) and itself was shaped by several Buddhist traditions including Vipassana, Zen Buddhism, and Vedanta (Kabat-Zinn, 2011; Sharf, 2015; Van Gordon et al., 2015). The second edition of the MBCT manual was substantially updated to reflect over a decade of subsequent clinical application and research, including findings from multiple controlled trials (Segal et al., 2013). MBCT aims to disrupt negative thinking patterns and cultivate present-moment awareness, cognitive flexibility and self-compassion. Like other first-generation MBIs, it presents mindfulness as a secular, attention-based program that largely omits Buddhist ethical or philosophical components (Van Gordon and Shonin, 2020; Zhou et al., 2024), leading to critiques that it oversimplifies traditional mindfulness (Francis et al., 2024; Krägeloh, 2013).

#### Second-generation Western integrations: MiCBT

Mindfulness-integrated cognitive behavior therapy is a second-generation MBI that responds to such critiques by integrating explicit Buddhist teachings within a secular clinical model (Cayoun et al., 2019; Francis et al., 2024). Emerging in Australia between 2001–2003, not long after the publication of the first MBCT trial, MiCBT draws on the Burmese Vipassana lineage of S. N. Goenka and systematically teaches the four foundations of mindfulness from the *Satipatthana Sutta* (Cayoun et al., 2019). Like other second-generation MBIs, such as Meditation Awareness Training (MAT; Van Gordon et al., 2014), Mindfulness-Based Interventions incorporating Compassion, Ethics, and Wisdom (MBI-CEW; Furnell et al., 2024), and Cognitively-Based Compassion Training (CBCT), MiCBT incorporates ethical and psycho-spiritual elements alongside Western cognitive-behavioral techniques.

A key aspect of MiCBT is the cultivation of non-reactive interoceptive awareness to foster emotional regulation and self-awareness. MiCBT functions as transdiagnostic intervention (Francis et al., 2024) and has demonstrated benefits across multiple domains including depression (Burgess et al., 2021; Pouyanfard et al., 2020; Sohrabi et al., 2022), health anxiety (Alipur et al., 2023), emotional regulation (Rasouli et al., 2023), substance use (Baker et al., 2014), and body image concerns (Rasouli et al., 2023). For a full review see Francis et al. (2024) and Boldt et al. (2025). Development began in 1989, with research commencing in 2001 and the first pilot study in 2003; later refinement led to its expansion from an 8-week to a 10-week program (Francis et al., 2024).

### Aim of the study

The aim of this study was to conduct a template analysis comparing the traditional applications of mindfulness, as described in the Satipaṭṭhāna Sutta, with its implementation in two independently developed and contrasting MBIs developed for psychological conditions, MBCT and MiCBT. MBCT was selected due to its status as a widely researched first-generation MBI, whereas MiCBT was chosen as a second-generation MBI for its explicit integration of traditional Buddhist principles. The Satipaṭṭhāna Sutta was used as the reference framework against which both interventions were examined.

The comparison evaluates differences in explicit theoretical and instructional frameworks rather than therapeutic effectiveness or clinical validity. Given the time-limited and clinically structured nature of typical 8-to-10-week interventions, MBIs would not be expected to replicate the full scope of classical Buddhist training. The analysis therefore focuses on what is explicitly represented within the intervention manuals when examined through a Buddhist psychological framework.

This comparison is intended to inform clinicians, researchers, and practitioners interested in deeper conceptual integration between traditional Buddhist psychology and contemporary mindfulness-based interventions.

## Methods

### Study design

This was a qualitative study using template analysis to systematically compare the traditional Buddhist text, the Satipaṭṭhāna Sutta, with two MBIs designed for psychological disorders: MBCT and MiCBT. Template analysis, as outlined by Brooks et al. (2015), provides a structured yet flexible framework that allows for categorization based on an initial coding framework while remaining open to the emergence of new themes from the data. The coding framework used in this study was derived from Anālayo’s (2003) breakdown of the Satipaṭṭhāna Sutta.

MBCT and MiCBT were selected for detailed comparison because both are structured, manualized interventions designed for mental health treatment that explicitly integrate mindfulness practice with cognitive-behavioral principles. MBCT (Segal et al., 2013) was chosen as a first-generation program with extensive empirical validation and widespread global use, providing a benchmark for Western, evidence-based mindfulness applications.

Mindfulness-integrated cognitive behavior therapy (Cayoun et al., 2019), in contrast, represents an MBI that reintroduces explicit Buddhist principles within a clinical framework, drawing conceptually from the Satipaṭṭhāna Sutta and Abhidhamma psychology rather than being derived from MBSR. This made it particularly relevant to the present study’s comparative framework.

While MBSR holds an important position in the wider MBI landscape, it was not included in the present comparison. The analysis focused on interventions whose manuals are structured around defined psychological disorder frameworks, allowing for a more controlled comparison of conceptual structure and instructional content. MBCT was designed as a relapse-prevention protocol for depression, while MiCBT targets transdiagnostic psychological disorders. By contrast, MBSR was originally developed as a stress-reduction program, later adapted for individuals with medical conditions such as chronic pain, and subsequently expanded to broader clinical and non-clinical populations (Francis et al., 2024; Van Gordon and Shonin, 2020). Its exclusion reflects the study’s focus on interventions whose manuals are structured around defined psychological disorder frameworks, rather than any assessment of its effectiveness or clinical relevance.

### Source materials

#### Buddhism texts

The Satipaṭṭhāna Sutta–translated by Anālayo (2003) is an early and foundational text that encompasses the teaching attributed to the Buddhist himself (Anālayo, 2003). It is found in the *Pali Canon* as part of the *Majjhima Nikaya* (Middle-Length Discourses), specifically as Sutta 10 and believed to have been composed in the 5th to 3rd century BCE, shortly after the Buddha’s lifetime (Anālayo, 2003). The text outlines a systemic practice of mindfulness. It composed of four contemplative foundations: the body, feelings, mind and Dhamma. The Sutta teaches the key Buddhist concepts of impermanence, dissatisfaction, and emptiness, fostering insight into the nature of reality.

#### Western MBIs texts

For readers seeking a concise session-by-session overview of MBCT and MiCBT, a comparative weekly breakdown is provided in Francis et al. (2024) (see Supplementary Table S1).

Mindfulness-Based Cognitive Therapy for Depression (Segal et al., 2013) is a manualized guide for practitioners implementing the MBCT program. First published in 2002, the book provides a comprehensive overview of the theoretical foundations of mindfulness and cognitive therapy, along with detailed instructions for implementing the therapy. MBCT is an 8-week program consisting of weekly group sessions and daily home practice. Each session is approximately two to two and a half hours long. It includes the following components: mindfulness meditation practices, cognitive, and some behavioral, techniques, group discussions, and homework assignments.

The Clinical Handbook of Mindfulness-Integrated Cognitive Behavior Therapy: a Step-By-Step Guide for Therapist (Cayoun et al., 2019) is a manualized guide for practitioners implementing MiCBT. It describes the principles and theoretical framework of MiCBT then provides detailed instructions for facilitating each two-hour session which consists of psycho-education, guided practice, discussions and homework (Cayoun et al., 2019). As part of the homework, participants are asked to engage in two 30-min meditation sessions a day. These meditations progress throughout the program starting from relaxation meditation, focusing on the breath to variations of body scanning. Guidelines are provided for delivery in either group or individual settings.

### Procedure

Although typically used for interview transcripts, this study applied template analysis to a traditional Buddhist text and to contemporary MBI manuals, offering a novel methodological approach (Brooks et al., 2015; King, 2012). The Satipaṭṭhāna Sutta was treated as an authoritative historical text, reflecting a *realist assumption* that it outlines an objective framework for mindfulness practice. In contrast, MBIs were analyzed through a *constructivist lens*, acknowledging that they have evolved within Western psychological paradigms rather than serving as direct iterations of Buddhist teachings.

Template analysis was chosen for its structured yet flexible approach, allowing for both deductive and inductive coding (Brooks et al., 2015; King, 2012). This method facilitated the identification of commonalities between the Satipaṭṭhāna Sutta and MBIs, as well as Buddhist principles that remain underutilized in contemporary mindfulness interventions.

Moreover, template analysis enhances transparency and replicability by providing a clear coding structure that can be validated across researchers (Brooks et al., 2015). This ensures methodological rigor when comparing texts from distinct epistemological traditions, offering valuable insights into how MBIs have adapted traditional mindfulness and what aspects of the Buddhist framework remain absent.

#### Template development and refinement

The primary researcher first familiarized himself with the base comparison text, the Satipaṭṭhāna Sutta, identifying four major categories based on its traditional breakdown (i.e., the Four Foundations of Mindfulness; Anālayo, 2003). Further categorization followed Anālayo’s (2003) 15-subcategory coding system, recognized in Buddhist scholarship and previous studies (Sedlmeier et al., 2023). These subcategories formed the initial template, which was then systematically compared against two selected MBI manuals to identify similarities and omissions. Given the conceptual differences between Buddhist and Western psychological frameworks, an iterative refinement process was adopted, allowing the coding framework to evolve as new insights emerged. For example, since the goals of the interventions differed, certain language and concepts required interpretation to align with either the modern psychological or traditional Buddhist framework. The interpretation for ‘suffering’, for example, proved complex and required ongoing adjustments. This process ensured that both traditional and contemporary perspectives on mindfulness were effectively represented and compared.

#### Data analysis: final coding and reliability

Each subcategory was initially rated by the primary researcher for similarity to the Satipaṭṭhāna Sutta using a seven-point Likert scale. The scale, adopted from Casper et al. (2020), was selected for its nuanced differentiation over a five-point scale (Aybek and Toraman, 2022).

To test the reliability of the coding process and ultimately improve the validity of the ratings (Cole, 2024), inter-rater reliability (IRR) was performed with two expert coders (Cole, 2024; Halpin, 2024; Gisev et al., 2013; Gwet, 2014) using the qualitative summaries of the subcategories of the Satipaṭṭhāna Sutta and the comparisons with the two MBIs (see qualitative findings below). The coders and primary researcher used the identical 7-point anchors as defined in Table 1. The validation was used to refine the coding framework and align interpretation of categories rather than enforce identical ratings, which aligns with standard qualitative reliability procedures (Gisev et al., 2013; Gwet, 2014). The two coders included a professor of psychiatry who had specific clinical training and substantial experience in cognitive-behavioral and mindfulness-based therapeutic interventions (along with extensive personal study in Buddhist and western approaches to understandings of consciousness), alongside a former Tibetan Geshe monk, translator, and researcher (PhD). Their combined expertise provided a balanced interpretation, integrating both traditional Buddhist and modern psychological perspectives. While variability in coder ratings may stem from this interdisciplinary lens, highlighting the complexities of bridging these frameworks, it enabled the revised final ratings to be grounded in broad perspectives.

**Table 1:** Initial and revised similarity ratings between the Satipaṭṭhāna Sutta, MBCT and MiCBT.

Foundation	Subcategory	Initial ratings		Revised ratings	
		MBCT	MiCBT	MBCT	MiCBT
Body	Breathing	6	6	6	6
	Posture	6	5	6	5
	Activities	6	5	6	5
	Anatomical Parts	1	1	1	1
	Elements	1	3	1	4
	Corpse in Decay	1	1	1	1
	Mean	**3.0**	**2.5**	**3.0**	**3.1**
Feelings	Affective Qualities	5	6	4	5
	Ethical qualities	2	5	2	4
	Mean	**3.5**	**5.5**	**3.0**	**4.5**
Mind	Ordinary States of Mind	2	4	3	4
	Higher States of Mind	1	1	1	1
	Mean	**1.5**	**2.5**	**2.0**	**2.5**
Dhamma	Hindrances	3	5	3	5
	Five Aggregates	2	3	2	3
	Sense-Spheres/Bases	1	2	2	2
	Awakening Factors	1	1	1	1
	Noble Truths	3	5	2	4
	Mean	**2**	**3.2**	**2.0**	**3**
Overall mean		**2.5**	**3.4**	**2.7**	**3.4**

1. Not at all similar (0%): The texts share no common themes, content, or meaning. 2. Little similarity (15%): The texts have minimal overlap, with only a few shared ideas or concepts. 3. Somewhat similar (25%): The texts share some common themes but differ significantly in details and presentation. 4. Moderately similar (50%): The texts have an equal balance of similarities and differences, sharing several key themes and ideas with some differences in details or emphasis. 5. Mostly similar (75%): The texts are alike in most aspects, sharing major themes and content, with minor differences. 6. Very similar (85%): The texts are highly alike, with only slight differences in wording or emphasis. 7. Completely similar (100%): The texts are nearly identical in meaning, content, and presentation, with no significant differences. Bolded values are the mean.

The coders participated in a one-hour training session, during which a subset of the data was pilot-coded before final coding. Discrepancies were resolved through discussion to refine the coding framework. A main rating session followed. After the main rating session, and to maximize the validity of the ratings, the researcher revised his original ratings based on the inputs from the two coders (Halpin, 2024; Gisev et al., 2013).

Inter-rater agreement across the three raters was assessed using two complementary approaches: Intraclass Correlation Coefficient (ICC) and Kendall’s W. Although Kendall’s W was originally planned as the primary measure of agreement, the use of Likert ratings resulted in many ties, limiting the informativeness of rank-based measures. Therefore, ICC was adopted as the primary analysis because it evaluates absolute agreement and is better suited for Likert ratings, which are treated as interval-level data. Kendall’s W (using a transposed orientation) is reported as a complementary measure of overall concordance.

All analyses were conducted in IBM SPSS Statistics (Version 30.0; IBM Corp, 2024), and significance was set at *p* < 0.05. ICC was calculated using a two-way random-effects model for absolute agreement, and average measures were computed. For Kendall’s W, the data were transposed so that judges were treated as cases (rows) and subcategories as variables (columns) to evaluate overall concordance in judges’ rating profiles. Confidence intervals for Kendall’s W are not available in closed form without bootstrapping (Gisev et al., 2013; Gwet, 2014); therefore, summary values are reported following accepted practice.

## Results

### Similarity ratings between the Satipaṭṭhāna Sutta, MBCT and MiCBT – quantitative findings

The initial and revised similarity ratings between the Satipaṭṭhāna Sutta, MBCT and MiCBT from the primary researcher are shown in Table 1. Although the initial ratings completed by the primary researcher showed high concordance with the other two raters, the differences between the initial and revised ratings suggests that additional calibration among raters and refinement of definitions contributed to greater consistency in evaluating the presence of Satipaṭṭhāna Sutta elements within MBCT and MiCBT.

This is also evident from the formal assessment of inter-rater agreement across the three raters. For the initial MBCT ratings, ICC was 0.98, 95% CI [0.95, 0.99], *p < 0.*001 and Kendall’s W was *W* = 0.88*, χ2*(14) = 37.10*, p < 0.*001. For initial MiCBT ratings, ICC was 0.94, 95% CI [0.87, 0.98], *p < 0.*001 and Kendall’s *W* was 0.91, *χ2*(14) = 38.10, *p < 0.*001. These findings indicate high consistency between the primary researcher and the two coders. Following refinements and clarifications of the coding scheme and subcategories through discussions amongst the raters to clarify category definitions and determine how specific MBI elements corresponded (or not) with the Satipaṭṭhāna Sutta, the revised ratings showed improved IRR. For final MBCT ratings, ICC was 0.99, 95% CI [0.96, 0.99], *p < 0.*001 and Kendall’s W was 0.91*, χ2*(14) = 38.01, *p < 0.*001. For final MiCBT ratings, ICC was 0.96, 95% CI [0.90, 0.99], *p < 0.*001 and Kendall’s *W* was 0.93, *χ2*(14) = 38.85, *p < 0.*001. These results indicate high concordance between raters, with further refinement of the coding scheme leading to increased reliability (Gisev et al., 2013; Gwet, 2014).

### Similarity between the Satipaṭṭhāna Sutta, MBCT and MiCBT – qualitative findings

At the start of each subcategory of the Satipaṭṭhāna Sutta there is a summary of the Satipaṭṭhāna Sutta to establish a foundational understanding of the traditional text. Following this, summaries of the comparison between this text and, MBCT and MiCBT are provided, including a discussion of the shared principles and distinct differences. Where significant differences arise, a more in-depth explanation of the Satipaṭṭhāna Sutta’s unique elements is provided.

### Mindfulness of body

#### Breathing

The Satipaṭṭhāna Sutta provides instructions regarding correct posture, observing changes in the breath and body as meditation progresses. It encourages cultivating continuous awareness of the breath to develop concentration and insight. The practitioner observes the natural flow of inhalation and exhalation without controlling it, noting whether the breath is long or short.

Both MBCT and MiCBT texts emphasize similar instructions, but how they deliver this information differ in their explicitness and approach.

**MBCT (6/7):** In MBCT, specific detailed guidance is provided on breathing techniques and different types of meditations (e.g., body scan meditation in Session 1 or mindfulness of breath in Session 2), whereas the Satipaṭṭhāna Sutta offers more implicit and poetic instructions that require more interpretation from the reader (e.g., ‘In regard to the body he abides contemplating the body internally, externally, and both.’; Anālayo, 2003, p. 5). MBCT places a great emphasis on placing awareness on the physical sensation of the breath, specifically the sensation of the abdomen rising/falling in their instructions (e.g., Session 2).

**MiCBT (6/7):** The instructions within the MiCBT handbook are both explicit and clear, mirroring the Satipaṭṭhāna Sutta in their discussion of correct posture and environment. They both provide detailed guidance on focusing on the breath, emphasizing the full body of the breath and the need for constant mindfulness, as instructions provide in Session 2 (Cayoun et al., 2019). The pacing aligns with the Satipaṭṭhāna Sutta, offering similar instructions for breath awareness such as, “Be aware of the breath coming in, going out … simple breath … breathe as you need to breathe, without controlling the breath” (Cayoun et al., 2019, p. 342). Additionally, the practice of sitting with minimized external stimulation is highlighted as similar to the Satipaṭṭhāna Sutta, which involves sitting quietly with eyes closed (optional) and without movement (Session 2; Cayoun et al., 2019).

#### Postures or physical states

The Satipaṭṭhāna Sutta emphasizes the importance of mindfulness across various physical postures, including walking, standing, sitting, and lying down, highlighting how mindfulness can be sustained in both active and passive states. The Sutta provides detailed guidance on maintaining awareness in each posture, stressing that mindfulness is not confined to a particular position but can be integrated into all aspects of movement and stillness. This adaptability allows the practice to permeate everyday life, encouraging continuous awareness of one’s actions and surroundings.

**MBCT (6/7):** MBCT shares a similar approach to postures, focusing on the importance of correct posture in enhancing meditation. MBCT suggests specific postures, such as sitting with a straight back and legs folded, to support stability and attentiveness during practice, as described in Mindfulness of Breath handout in Session 2 (Segal et al., 2013, p. 171). Like the Satipaṭṭhāna Sutta, in sessions and homework, MBCT emphasises that mindfulness can be cultivated across a variety of postures, from more dynamic movements like walking or simple yoga to more restful states, making mindfulness adaptable and relevant in various contexts of daily life.

**MiCBT (5/7):** MiCBT also incorporates the use of postures but in a more structured manner compared to the Satipaṭṭhāna Sutta. While the Sutta promotes mindfulness integration into all daily activities, MiCBT focuses on designated practice sessions, encouraging participants to engage in mindfulness exercises within a controlled environment and largely only in a sitting posture. The structured approach of MiCBT aims to minimize distractions and gradually build concentration, whereas the Satipaṭṭhāna Sutta encourages an ongoing, real-time awareness of one’s thoughts, feelings, and bodily sensations, regardless of the activity or posture. This is present in MiCBT, but not to the same extent emphasized in the Sutta.

#### Activities

Satipaṭṭhāna Sutta emphasizes the integration of mindfulness into all daily activities, encouraging continuous awareness across all actions. This structured and repetitive approach serves as a constant reminder to incorporate mindfulness into all aspects of life, highlighting its transformative potential when consistently applied to everyday actions. The Sutta’s approach fosters a persistent state of mindfulness that enhances mental clarity, well-being, and connection to the present moment.

**MBCT (6/7):** Like the Satipaṭṭhāna Sutta, MBCT emphasizes the significance of integrating mindfulness into daily activities, encouraging practitioners to maintain presence and awareness throughout all actions, whether during formal meditation or in the routine tasks of everyday life. MBCT specifically focuses on cultivating mindfulness by remaining present and avoiding attachment to wandering thoughts during ordinary activities, such as in exercises like the raisin activity (Session 1). The Satipaṭṭhāna Sutta reinforces this concept through a more structured and repetitive approach, instructing practitioners to act with clear awareness in various scenarios (e.g., “when a monk does X, he acts clearly knowing.” Anālayo, 2003, p. 19). Both approaches highlight the transformative potential of mindfulness when consistently applied to all actions, fostering a connection to the present moment and enhancing overall mental clarity and well-being.

**MiCBT (5/7):** Unlike the Satipaṭṭhāna Sutta, which recommends mindfulness during any activity, MiCBT advocates a more structured practice schedule which however incorporates mindfulness in daily activities in line with that structure and through the use of exposure and communication exercises.

Sessions evolve weekly, starting with basic practices, which include body relaxation and mindfulness of the breath meditation, and then advancing to more progressively refined body scanning meditations.

The Sutta advocates for integrating mindfulness into all aspects of experience, from mundane tasks to more significant actions whereas MiCBT employs a more structured approach by training the mind to reduce distractions, focusing on meta-cognitive awareness, equanimity and the co-emergence model. MiCBT suggests that the practice becomes easier and quicker with its eventual aim to be incorporated into daily life. MiCBT prioritises structured sessions designed to manage and reduce emotional responses and presents mindfulness more scientifically and methodologically. In contrast to the broader spiritual framework of the Satipaṭṭhāna Sutta, MiCBT pairs mindfulness practice with Western behavioral approaches to increase mindfulness in daily life, while the Satipaṭṭhāna Sutta advocates for persistent mindfulness across a wide range of daily activities.

#### Anatomical parts, elements and corpse in decay

Satipaṭṭhāna Sutta places an emphasis on understanding the body through its anatomical parts and elements, such as bones, organs, and other body parts that are typically seen as unpleasant. This approach is intended to act as an antidote to the attachment of the physical form by illustrating the body’s repulsive nature. The Sutta extends this exploration to include contemplation of the body in various stages of decay, emphasizing the impermanence of physical beauty and fostering detachment and insight into the nature of suffering.

MBCT–Anatomical parts (1/7), Elements (1/7), and Corpse in decay (1/7): Anatomical parts, elements and corpses in decay are a significant focus in the Satipaṭṭhāna Sutta but are not covered explicitly in MBCT.

MiCBT–Anatomical parts (1/7), Elements (4/7), and Corpse in decay (1/7): MiCBT and the Satipaṭṭhāna Sutta approach the use of the body and the four elements differently. While, MiCBT does not use the body, its constituents and the cycle of decay as a method to disassociate from the self-concept, it does make use of the four elements, albeit with a different emphasis. MiCBT discusses how Buddhism views the world as composed of four basic elements: earth, air, fire, and water (Cayoun et al., 2019, p. 119). It uses these elements to draw analogies to how we experience emotions in the body through interoceptive concomitants, such as a sense of mass (earth), motion (wind), temperature (fire), and fluidity (water). MiCBT uses the four elements as a framework to categorize and mindfully assess the quality of one’s feelings, recording them on the Mindfulness-based Interoceptive Signature Scale (MISS) for this purpose. This differs from how the Satipaṭṭhāna Sutta uses the elements to deconstruct the body into parts, highlight the suffering of impermanence and attachment to an unchanging self. While there may be an analogy in how MiCBT uses this theory to deconstruct the transient nature of one’s feelings, the underlying lesson and application are not the same.

### Mindfulness of feelings

#### Affective qualities

Satipaṭṭhāna Sutta offers a comprehensive exploration of affective qualities, categorizing feelings into pleasant, neutral, and unpleasant. The Sutta describes how these feelings arise, their impact on the mind, and their role in suffering. It emphasizes mindfulness as a tool to observe these feelings without attachment or aversion, thus fostering equanimity. The Sutta encourages practitioners to understand the cause of feelings and the broader implications for mental and spiritual well-being.

**MBCT (4*/7*):** MBCT shares similarities with the Satipaṭṭhāna Sutta in its categorization of affective experiences into pleasant, neutral, and unpleasant. However, MBCT primarily targets negative feelings associated with depression, often focusing on how negative thinking patterns can perpetuate suffering. In MBCT, sessions 1 and 2 introduce the connection between thoughts and feelings, with an emphasis on negative reactions to events. Session 4 briefly touches on the recognition of pleasant, unpleasant, and neutral feelings, but the session predominantly targets negative affect associated with depression.

While MBCT introduces the concept of the connection between thoughts and feelings, it places a stronger emphasis on mitigating negative affect, offering strategies to interrupt the automatic labelling of negative stimuli. Although Session 5 addresses observing and accepting emotions, as well as pleasant and neutral feelings, the primary goal of MBCT is to alleviate depressive symptoms, rather than to offer a comprehensive exploration of all affective states and strategies for managing them. The Satipaṭṭhāna Sutta provides specific instructions for dealing with emotional extremes, including antidotes for unwholesome mental states, which are less explicitly addressed in MBCT.

**MiCBT (5/7):** MiCBT closely mirrors the Sutta’s approach to affective qualities, discussing pleasant, neutral, and unpleasant feelings in a detailed and structured manner. In Session 3, MiCBT discusses the causes of suffering, unpleasant feelings and the effects of labelling stimuli or situations. This conversation is emphasized in Session 4’s Q&A section. Furthermore, Session 5 addresses the consequences of avoiding unpleasant experiences, noting how this behavior can lead to depression, missed opportunities, or failure. In Session 6, there is a chapter dedicated to cravings and aversions, detailing how the labelling of sensations significantly impacts our experience.

MiCBT emphasizes understanding the causes of suffering and how labelling experiences influences emotional responses, similar to the Sutta’s teachings on the impact of interpretation on suffering. The program integrates breath awareness and body scanning techniques to cultivate a state of equanimity, helping individuals disidentify from thoughts and adopt a more objective stance toward their feelings (e.g., ‘bipolar exposure’, a MiCBT term referring to equanimous exposure to both pleasant and unpleasant emotional states). This concept is explored in Session 6, which discusses cultivating equanimity towards experiences. MiCBT’s structured approach is rooted in the co-emergence model, which aligns with the Sutta’s focus on maintaining balance across all emotional states. However, MiCBT remains a therapeutic intervention compared to the philosophical and spiritual context of the Satipaṭṭhāna Sutta.

#### Ethical qualities

Satipaṭṭhāna Sutta incorporates a nuanced understanding of ethical qualities by categorizing feelings into two subcategories: “worldly” and “unworldly.” In this context, “ethical” refers to the functional role of mental states in supporting or obstructing progress, rather than to moral judgement or religious belief. Worldly feelings are those associated with physical sensations derived from the five senses, whereas unworldly feelings pertain to experiences aligned with spiritual practice or renunciation beyond worldly concerns (Anālayo, 2003). This categorization reflects the ethical context of a feeling’s origin rather than its affective nature, offering a more complex framework that evaluates whether a feeling propels or hinders spiritual progress. For example, guilt from neglecting meditation, though associated with an unpleasant feeling tone, may nonetheless motivate spiritual advancement by prompting renewed engagement with practice, while excessive joy following a successful meditation session, though associated with a pleasant feeling tone, could foster attachment to the meditation outcome and impede future progress through distraction and craving.

**MBCT (2/7):** This distinction between worldly and unworldly feelings is absent in MBCT, which lacks an explicit spiritual context, as would be expected given its secular therapeutic aims. An analogy could be made with MBCT assessing whether feelings contribute to or detract from positive mental health and well-being. Worldly or unworldly feelings, like ones arising from spiritual practice or dhamma adherence in the Satipaṭṭhāna Sutta, could align with feelings fostering positive mental health or progress in MBCT.

**MiCBT (4/7):** While the Satipaṭṭhāna Sutta categorizes feelings as worldly or unworldly, MiCBT uses a different framework, categorizing thoughts and emotions as ‘helpful’ or ‘unhelpful’ (Cayoun et al., 2019, p. 65) in relation to psychological distress and reactivity and that we often try and avoid unpleasant thoughts. While MiCBT’s framework is more aligned with Western psychological concepts, it shares some similarities with the Satipaṭṭhāna Sutta by focusing on the feelings when we try to advance or impede well-being. For instance, MiCBT emphasizes reducing avoidance and increasing desensitization when we try and avoid painful activities, even if they could be beneficial (e.g., *Handout 6.4, The Generalizing Effects of Avoidance and Desensitization*; Cayoun et al., 2019). Though MiCBT’s emphasis is on avoidance or abstaining from, the underlying principle aligns with the Satipaṭṭhāna Sutta’s assessment of actions that either align with or deviate from the spiritual path.

### Mindfulness of mind

The Satipaṭṭhāna Sutta discusses ordinary and higher states of mind. These states are categorized based on whether they help or hinder the path to enlightenment. The Sutta also discusses antidotes to unwholesome or unhelpful states of mind. Contemplating one’s state of mind is suggested as a method to detach from that state or to enhance it, similar to the approach in the Feeling Foundation. While feeling tone and states of mind are closely related, the Sattipaṭṭhāna Sutta treats them as analytically distinct phenomena, with mindfulness of feelings concerned with hedonic tone and mindfulness of mind concerned with the qualities and trajectories of mental states that arise in response to that feeling tone.

#### Ordinary states of mind

**MBCT (3/7):** MBCT does not address states of mind within an ethical or spiritual framework. Instead, it focuses on identifying and reframing negative cognitive and emotional patterns that contribute to conditions of depression and anxiety. MBCT encourages individuals to observe their thoughts and feelings without judgement, fostering metacognitive awareness and acceptance rather than prescribing antidotes for unwholesome states (Shapiro et al., 2006; Sipe and Eisendrath, 2012). The absence of a direct spiritual or ethical context in MBCT reflects its foundation in Western psychological models, which prioritize disassociation and cognitive restructuring over the ethical or spiritual transformation.

**MiCBT (4/7):** In the Sutta, mental states are categorized as either ordinary or higher, depending on whether they hinder or help the path to enlightenment. In MiCBT, this concept is implicitly addressed in sessions eight and nine, though not explicitly in relation to Buddhism but rather in the context of self-help and interpersonal skills. MiCBT explains how some states of mind can be helpful or hindering to a person. For example, these sessions discuss how certain states of mind, such as anger, are unhelpful when resolving issues. MiCBT highlights that interpreting events and people as right or wrong and reacting with anger is counterproductive. In Session 9, MiCBT also discusses ethical actions, noting that “Understandably, doing harm to oneself or to others is likely to increase distress caused by the act itself, by subsequent guilt and self-blame, and by the possible interpersonal conflicts caused by the action.” (Cayoun et al., 2019, p. 287).

The Sutta suggests that contemplating one’s state of mind can help in either detaching from or enhancing that state and subsequent reaction, similar to its approach in the Feeling Foundation. MiCBT, similarly, emphasizes that any learned behavior can be modified or reconditioned. For instance, Session 8 on assertiveness training and Session 3 on exploring the meaning and beliefs associated with events illustrate how individuals can make choices and learn to reshape their responses to stimuli.

#### Higher states of mind

The concept of the “Great” state of mind in Buddhist practice refers to the ability to expand one’s object of meditation in all directions and is closely associated with the development of deep meditative absorption. This advanced meditative state involves cultivating a boundless, expansive awareness that encompasses all phenomena without attachment.

**MBCT (1/7) and MiCBT (1/7):** MBCT and MiCBT do not discuss this concept, likely because it is a specific belief and practice within higher-level aspects of Buddhist philosophy that focus on achieving profound meditative absorption.

### Mindfulness of Dhamma

#### Five hindrances

The five hindrances represent mental states that obstruct the clarity and insight essential for effective meditation practice. These hindrances, when present, impede the mind’s capacity to achieve deep concentration. They include *sensual desire*, which manifests as craving for sensory gratification; *aversion*, characterized by avoidance and rejection towards unpleasant experiences; *sloth and torpor*, a state of dullness or lethargy that hampers mental clarity; *restlessness and worry*, marked by agitation and distraction; and *doubt*, which undermines confidence and trust in the process.

Each hindrance poses a challenge to meditative progress, but antidotes exist to counteract their influence. Failure to recognize the presence of hindrances may lead to ineffective meditation, as individuals may find themselves trapped in cycles of distraction or mental turmoil.

**MBCT (3/7):** While discussions on general hindrances in meditation are present within MBCT, specific guidance on addressing the Five Hindrances in Buddhism are not explicitly covered in MBCT. While MBCT addresses common challenges encountered during meditation practice through the inquiry process in the handbook, it depends on what is raised by participants and may not offer comprehensive strategies for managing each of the five hindrances.

**MiCBT (5/7):** MiCBT includes discourse on these hindrances in sessions three, six and seven. For instance, it discusses agitation and its antidotes to meditation (Cayoun et al., 2019, p. 136–137) and addresses related questions in the Q&A sections about meditation difficulties (Cayoun et al., 2019, p. 216). Additionally, MiCBT elaborates on how to recognize the hindrances, what leads to their presence, and which antidotes to apply in the mentioned sessions.

#### Five aggregates

The concept of the Five Aggregates serves as a foundational aspect of Buddhist teachings aimed at dismantling the illusion of a permanent and unchanging self. Through introspection and observation, practitioners engage in a contemplative exploration of the components that constitute the sense of self. These aggregates are understood as transient and impermanent, emphasizing the reliant nature of existence.

The five aggregates include *form*, encompassing the material qualities of matter, including one’s physical body and the external world; *feeling*, which arises in response to sensory stimulation; *cognition or discernment*, involving the interpretation and categorisation of sensory data; *volition or intention*, directing purposeful action; and *consciousness*, the awareness of mental and sensory experiences (Anālayo, 2003).

By recognizing the continual flux and impermanence inherent in these aggregates, individuals can begin to loosen their attachment to the notion of a fixed self-concept, such as a soul or ‘I’. Buddhism questions the existence of an ‘I’ if it is dependent on changing parts to exist (Anālayo, 2003).

**MBCT (2/7):** In MBCT, explicit discussion of the Five Aggregates is absent, but analogous exploration occurs when MBCT examines feelings and fosters non-attachment thinking. While MBCT acknowledges feelings as transient phenomena, it may not directly frame them as aggregates or permanent aspects of the self but emphasizes a detachment from them as they are not to be considered part of the self or ultimate truths (e.g., Session 6). This might not be explicitly or as philosophically existential, but a theme of non-self is still present.

**MiCBT (3/7):** The same is similar for how MiCBT relate to feelings. Additionally, MiCBT references the Five Aggregates in its co-emergence model, using them to illustrate the characteristics of how consciousness and the mind operate. However, this interpretation differs from the way the Satipaṭṭhāna Sutta employs the aggregates, which focus on disassociating from the sense of self. In MiCBT, the aggregates serve more as a framework for understanding mental processes than as tools for transcending self-identification.

#### Sense sphere or bases

The sense-bases consist of the sense organs and their objects. This includes the physical senses (i.e., ear, eye, nose, tongue, and body) and their respective objects (i.e., sounds, sights, smells, tastes, and touches). In Buddhist psychology, each sense base is associated with its own mode of consciousness, with the mind considered the sixth sense, with its mental objects. This framework differs from most Western psychological models, which typically assume a unified consciousness processing multisensory input.

Contemplation on the Six Sense-Bases can help illuminate personal biases and tendencies in the process of perception, facilitated by mindfulness. When unconscious habituations and incorrect views become conscious, one can apply antidotes to unwholesome views.

Cognizing something is the culmination of past conditioning and present mental inclinations, determining which particular parts of the object stand out during cognition. Reflective or analytic thought enters the process afterward, based on the parts that stood out from the initial cognitive process. Through mental training, one can habituate new patterns and ultimately change cognition.

By repeatedly focusing awareness, a familiarity with this type of thinking will imprint on how one views experiences, leading to similar ways of cognition in the future. This gradual habituation transforms the way the world is recognized and can impact what one perceives as agreeable/attractive or disagreeable/aversive.

For example, one could focus on the sense basis of hearing and sound with the negative labelling that arises when hearing someone chewing loudly. Through mindfulness, one can first become aware of the aversion to the sound and then work to restructure the response, moving from a reactive aversion to a more balanced and non-judgmental awareness of the sound.

**MBCT (2/7):** As a specifically Buddhist philosophical concept, cognition in this context is not present in MBCT. While an argument could be made that certain philosophical elements are indirectly present, such as focusing on aspects of some sensory spheres (e.g., the sensation of touch or feeling during the breath) and the thoughts or sensations that arise alongside them. However, these aspects are not framed or presented in the same way as its traditional Buddhist approach (e.g., each sense having its own consciousness). The only similarity would be that there are five senses and their categorical similarity.

***MiCBT* (2/7):** MiCBT discusses the Sense-Bases, although with different details and conceptualizations compared to traditional Buddhist teachings. While the Satipaṭṭhāna Sutta presents the Sense-Bases as a means of understanding perception and mental processes, MiCBT focuses on how sensory experiences can be influenced by triggers from emotional responses. This is similar to how Buddhism addresses conditioning, sensory responses, and habitual patterns, but MiCBT does so from a psychological framework that does not necessarily incorporate the broader Buddhist understanding of cognition and the path to liberation.

#### Seven requisites of enlightenment or awakening factors

The Seven Requisites of Enlightenment are seven stages or factors one needs to reach enlightenment. The instructions on the awakening factors follow a similar pattern to the teachings on the Five Hindrances, providing commentary on the presence or absence of each quality and the conditions conducive to its presence or absence. These seven factors can be categorized into two distinct groups, with mindfulness serving as the overarching aspect that enhances awareness and facilitates their development. The remaining six factors can be further divided to address the needs of either a sluggish or over-energetic mind. For individuals experiencing a *lack of mental energy or vitality*, practices such as investigation-of-dhamma, energy, and joy can be beneficial. Conversely, for those grappling with an *overly active or agitated mind*, cultivating tranquillity, concentration, and equanimity can offer support and balance.

**MBCT (1/7) and MiCBT (1/7):** This framework is not covered by MBCT or MiCBT, as it does not include these specific teachings. Even though a core aspect of MBCT and MiCBT is cultivating aspects such as tranquillity and equanimity it is not presenting in the same philosophical backdrop.

**MBCT (1/7) and MiCBT (1/7):** The seven awakening factors as an integrated framework are not explicitly taught within MBCT or MiCBT manuals. While both programs cultivate qualities such as tranquillity, concentration, and equanimity, these are not presented within the diagnostic and regulatory structure of the awakening factors, which links specific qualities to particular mental imbalances (e.g., sluggishness versus agitation). As such, these qualities appear in MBCT and MiCBT in a decontextualized form, rather than as components of the broader Buddhist framework described in the Sattipaṭṭhāna Sutta.

#### Four Noble truths

The Four Noble Truths serve as a cornerstone of Buddhist philosophy and are integral to understanding the role of mindfulness in alleviating suffering. *Firstly*, they elucidate that life is inherently characterized by dissatisfaction and suffering. *Secondly*, they identify the cause of suffering as karma and affliction, that include cravings or aversions to objects or situations deemed desirable. *Thirdly*, they posit that the cessation of suffering can be achieved through enlightenment. *Lastly*, they prescribe the Noble Eightfold Path as the means to attain enlightenment, comprising three key components: Wisdom (right view and right intention), Ethics (right speech, right action, and right livelihood), and Concentration (right effort, right mindfulness, and right concentration). In the context of mindfulness, these truths underscore the importance of cultivating awareness of suffering and its causes, as well as understanding the path to liberation. Mindfulness practice facilitates this by enabling individuals to observe their experiences with clarity and discernment, ultimately leading to a deeper comprehension of reality and the potential for liberation from suffering.

**MBCT (2/7):** MBCT incorporates some teachings related to the Four Noble Truths, emphasizing the significance of mindfulness in recognizing the nature of suffering and realizing a way to alleviate it. While MBCT does not explicitly reference the Buddhist path to enlightenment, it shares the practical goal of reducing suffering, particularly in the context of depression (e.g., Session 5). However, while MBCT aligns with this principle in helping individuals gain insight into their thought patterns, it does so within a psychological framework rather than the broader spiritual and ethical context in Buddhism.

**MiCBT (4/7):** MiCBT explicitly explains these truths in several sessions. For example, in Session 7, an anecdote about an officer is used to explain the Four Noble Truths. Additionally, Session 9 discusses why individuals suffer, particularly focusing on the first truth, dukkha, when addressing compassion and the ego. The fourth Noble Truth is partially covered through discussions of the Eight-Fold Path, such as harmful speech, taking lives, and taking what is not given, in Session 9. It can also be argued that Buddhist teachings in general are integrated throughout MiCBT, reflecting its foundational influence on the program.

## Discussion

Overall, the results indicate that both MBCT and MiCBT demonstrate the strongest alignment with the first two foundations of the Satipaṭṭhāna Sutta: mindfulness of body and mindfulness of feelings. Both interventions emphasize attention to breath, posture, activity and affective experience, supporting the development of concentration and equanimity. However, as the Satipaṭṭhāna Sutta moves into more cognitive, ethical and philosophical aspects, alignment with Western interventions decreases. This divergence is more pronounced in MBCT than in MiCBT, the latter being more explicitly grounded in Buddhist teachings and four foundations of the Satipaṭṭhāna Sutta (Francis et al., 2024). Aspects of the Satipaṭṭhāna Sutta with limited representation in these interventions may offer opportunities for further development. Accordingly, this discussion first examines areas of overlap before considering elements of traditional Buddhist practice that may extend contemporary MBIs.

### Mindfulness of the body

The body-focused foundation of the Satipaṭṭhāna Sutta grounds practitioners in physical experience, cultivating a core meditative skill: focused attention (Anālayo, 2003; Thanissaro and DeGraff, 2012). By directing mindfulness to the body, it provides a foundation for introspection and analysis meditations. This practice invites contemplation of existential questions, such as the impermanence of the body. The revised similarity rating results demonstrated that it was the equally highest-rated foundation in MBCT (equal with feeling foundation) and it was the second highest for MiCBT.

#### Breathing, activities and postures

Both MBCT and MiCBT align with breathing, activities and postures contemplations of the Satipaṭṭhāna Sutta but differ in their approach and emphasis. While both programs are experiential, MBCT’s format is broader and more eclectic, integrating varied practices and cognitive strategies (e.g., the raisin activity and three-minute breathing space). MiCBT offers a more scaffolded training progression that mirrors aspects of traditional contemplative practice, where stability in breath awareness supports deeper insight into reactivity, intention, and behavior change. Rather than suggesting that one approach is superior, these distinctions highlight different therapeutic pathways: MBCT emphasizes experiential learning across varied life domains, whereas MiCBT builds mindful awareness through structured, cumulative training. Future research could investigate how these different approaches to breath-based mindfulness influence clinical outcomes, especially in terms of depth of insight, emotional regulation, and sustained behavioral change.

#### Anatomical parts and elements

The Satipaṭṭhāna Sutta emphasizes the dissolution of attachment to the self through contemplations on the body’s composite and impermanent nature, including meditations on anatomical parts, elements, and the decay process. This approach aims to reduce attachment to the permanent unchanging physical self (Anālayo, 2003; Thanissaro and DeGraff, 2012). While MBCT omits using anatomical parts or elements, MiCBT includes the elements but not for the purpose of considering the cycle of death and decay or detaching from an unchanging-self.

Incorporating reflections on bodily decomposition into future iterations of MBIs could help individuals loosen ridged self-concepts and associated suffering. This practice aligns with the Buddhist aim of fostering an understanding of impermanence and reducing attachment to the self, potentially enhancing the positive mental health effects of MBIs. Research suggests that contemplating death and dying may offer psychological benefits (Harpham, 2014; Kashdan et al., 2014; Rushton et al., 2009). However, further investigation is needed to determine whether this practice poses risks for individuals with suicidal ideation or conditions such as body dysmorphic disorder.

### Mindfulness of feelings

The Satipaṭṭhāna Sutta categorizes feeling tone as pleasant, neutral, and unpleasant, and emphasizes mindfulness observation without attachment or aversion (Anālayo, 2003; Feldmann, 2016). The similarity rating results demonstrated that the feeling foundation was the highest rated foundation in MBCT (equal with body foundation) and highest for MiCBT. MBCT similarly identifies affective states but concentrates more on mitigating negative feelings, particularly those associated with depression. MiCBT, like the Sutta, focuses on understanding and disidentifying from all categorizations of feelings with a similar structure in their co-emergence model to how the Satipaṭṭhāna Sutta describes feeling tone. This is further described below.

Regarding ethical qualities, the Satipaṭṭhāna Sutta differentiates between “worldly” and “unworldly” feelings, highlighting their spiritual implications. MBCT lacks this spiritual distinction but shares a similar approach to detachment from feelings, promoting mental health rather than spiritual progress. MiCBT uses a practical distinction between “helpful” and “unhelpful” thoughts and emotions, focusing on cultivating equanimity and detachment from emotional extremes, much like the Satipaṭṭhāna Sutta.

#### Feeling tone and pleasant, unpleasant, or neutral perception

In Buddhism, *Feeling* is a specific term with a specific role in the functioning of mind and consciousness (Feldmann, 2016; Thanissaro and DeGraff, 2012). The mind is conceptualised through 51 or 52 mental factors, depending on the tradition (Feldmann, 2016). Each ‘factor’ or aspect of the mind has a specific function when generating consciousness moment-to-moment. *Feeling, or ‘vedanā’* in Pali, is one mental factor that plays a crucial role in experiencing objects perceived by the six consciousnesses (Batchelor, 2019; Feldmann, 2016; Thanissaro and DeGraff, 2012).

The six-sense consciousness’ are similar to the five senses in the West but in Buddhism each sense has its own consciousness. For example, the *eye consciousness* perceives visible objects, the *tongue consciousness* perceives taste, and so on. The five-sense consciousness’ are responsible for categorizing the initial experience from the senses as pleasant, unpleasant, or neutral. For example, the body consciousness perceives the sensation of pain from stepping on a Lego piece as an unpleasant feeling. The word “feeling” does not refer to emotions, as often use it in the West, but to the bare affective tone of experience, whether pleasant, painful, or neutral (for further review see: Batchelor, 2019).

There is also another overarching, sixth consciousness, called the *mental consciousness,* that may be either perception or conception. Mental conception engages sensory information from the five sense consciousnesses, processes this information and is associated with thoughts regarding the ego, ‘I’, ‘the witness’ and so on. This will be further discussed in the mind foundation below.

#### The potential benefits of contemplating the feeling foundation

Contemplating the feelings foundation helps prevent these tones from escalating into craving or aversion and allows for the reassessment of already labelled tones (Batchelor, 2019). By reassessing the experience as pleasant, unpleasant, or neutral one can eventually scale back some of the labels attached to the sensation and revert it back to the bare tone. More accurate appraisal of feeling tone helps reduce the suffering associated with unmet expectations.

Mindfulness practice may reduce discomfort associated with remaining in one position for prolonged periods by altering how sensation is appraised. Attending to bodily sensation and recognizing its associated unpleasant feeling tone, often labelled as “painful,” allows for a more objective reassessment of intensity and may reduce associated suffering (Bodhi, 2023). This mechanism has been observed in mindfulness-based interventions for chronic pain, including Mindfulness-Based Stress Reduction (Soundararajan et al., 2022).

Batchelor (2019) argues that sustained attention to the initial sensory experience, or “bare tone,” interrupts habitual attachment and aversion before cognitive elaboration occurs. By responding at the level of feeling tone, mindfulness can limit escalation into negative thought patterns without relying on cognitive restructuring.

Feeling tone/vedanā has already gained some traction as an adjuvant to MBI’s. A recent 8-week follow-on trial from a mindfulness course focusing on the vedanā showed significant reductions in depression, stress, anxiety, increases in well-being and mindfulness (Williams et al., 2022). This demonstrates the potential for follow-up interventions using more specific Buddhist theories, like Vedanā, centered on individuals with prior mindfulness experience (Williams et al., 2022). Whether this study would add value above and beyond other programs that implicitly incorporate Vedanā, like MiCBT with its interoception focus, is an area for further investigation.

### Mindfulness of mind

It is important to clarify that MBIs, like MBCT and MiCBT, contain substantial cognitive content, including decentering, metacognitive awareness, and recognition of cognitive patterns. The present analysis does not suggest these processes are absent. Rather, the comparison examines how they are conceptualized when viewed through a Buddhist psychological taxonomy. In this framework, the *mindfulness of mind* refers not to cognition broadly, but to the identification of mental states and their qualities. The differences observed therefore reflect distinctions in theoretical organization rather than a lack of cognitive emphasis within MBCT.

Accordingly, an intervention may engage extensively with affective, cognitive, and behavioral processes, including pre-conceptual reactivity, yet still differ from the Sutta’s mindfulness of mind foundation, which organizes these processes in terms of their ethical qualities. When examined using this definition, both MBCT and MiCBT showed limited explicit integration of the mind foundation. Similarity ratings indicated that it was the lowest-rated foundation in MiCBT and equally lowest in MBCT (alongside the Dhamma foundation). The purpose of contemplating the mind foundation is to encourage practitioners to observe and understand the nature of their thoughts, emotions, and the mental states that give rise to them (Thanissaro and DeGraff, 2012). The emphasis on the body and feelings foundations in MBCT and MiCBT, rather than the mind foundation, may be due to the added complexity of this approach, potential resistance to deeper Buddhist philosophical concepts, and hesitancy to introduce evaluative judgements about mental states within the non-judgmental framework of Western psychology. The following section explores the Buddhist perspective on consciousness, states of awareness, and how these concepts could be adapted for use in Western psychology and MBIs.

#### The sixth consciousness–mental consciousness

*Mental consciousness* is responsible for the awareness of an object within any given moment of consciousness (Feldmann, 2016; Gyel-tsen, 2003). Unlike *sensory consciousnesses*, which simply perceive external stimuli, *mental consciousness* extends beyond mere perception by interpreting, conceptualizing, and assigning meaning to experiences. It is responsible for conceptualizing the self and sometimes could be conceived as the “self” or the “witness,” depending on the lineage or framework. According to Buddhist psychology, mental consciousness provides the perceptual and contextual expansion of a feeling tone, shaping how experiences are understood and responded to Feldmann (2016) and Gyel-tsen (2003). It cognizes mental phenomena, including thoughts, emotions, memories, and mental images, and plays a crucial role in interpretation and evaluation.

#### A moment of awareness in Buddhism

A moment of consciousness, according to Buddhism, often begins when a *sensory consciousness* encounters an external object, and to a perception of a *feeling tone* as pleasant, unpleasant, or neutral (Feldmann, 2016; Gyel-tsen, 2003). *Mental consciousness* then processes this initial feeling tone, expanding upon it and engaging with various mental factors that further shape the experience. So, to further develop the example above, stepping on a Lego piece initially generates a painful sensation experienced as unpleasant by the *sense consciousness*. However, *mental consciousness* does not merely register the pain; it engages with additional mental factors that shape the experience further. Mental factors like *attachment* to comfort and *aversion* to pain may amplify distress, while *anger* or *resentment* toward the person who left the Lego piece on the floor could arise.

Mental factors emerge almost simultaneously with the initial perception, shaping how one conceptualizes and reacts to an experience, ultimately leading to different mental states (Gyel-tsen, 2003). These responses are influenced by past tendencies and contextual factors. Through mindfulness, Buddhist practice encourages the recognition of these mental factors, allowing individuals to disengage from reactive patterns and cultivate greater clarity and equanimity in response to experiences.

#### A mind that possesses states

In Buddhism, and outlined in the Satipaṭṭhāna Sutta, the mind possesses mental states or factors, such as anger, that arise and pass away, rather than defining the person. A person’s mind is therefore said to *have* anger or joy, much like a person’s liver could have an illness, rather than *be* angry or joyful. This is somewhat analogous to distinctions made in contemporary psychology between temporary mental states and more enduring tendencies (Davidson, 2010), where repeated cultivation of particular states can gradually shape longer-term patterns of experience. In Buddhist psychology, however, these long-term patterns are not viewed as stable personality traits, but as conditioned mental habits shaped through practice. Through repeated cultivation of wholesome states, enduring beneficial tendencies can develop while unwholesome states can be reduced or even eliminated over time (Anālayo, 2003; Feldmann, 2016).

Like the classification of feeling tones, Buddhist mental factors are categorised into three main subcategories (Feldmann, 2016; Gyel-tsen, 2003). These include *virtuous*, *non-virtuous*, and *neutral* factors. *Virtuous* factors support mental clarity, ethical conduct, and progress on the path to enlightenment, while *non-virtuous* factors contribute to suffering and reinforce delusions. *Neutral* factors are intrinsically neither beneficial nor harmful; their effects depends on how they interact with other mental states.

A key tenant in Buddhist psychology is that the mind is naturally free from negative non-virtuous mental factors and fundamentally only possesses virtuous or neutral mental factors (Seegers, 2024). Buddhist teachings often describe the mind as inherently clear, likened to water that may become muddied but can return to clarity (Gilbert et al., 2024). So, with practice, Buddhists believe that negative/afflicted mental states can be recognized, detached from, and ultimately removed with antidotes (Feldmann, 2016; Gyel-tsen, 2003). For example, compassion (non-violence) is prescribed as a countermeasure to *anger*, and while *wisdom* counters *ignorance* (afflicted view).

#### Contrast with Western psychological models

Western psychological models, including CBT and mindfulness-based interventions, distinguish thoughts, emotions, and behaviours and aim to help individuals observe and modify these processes. Mindfulness practices within MBIs strengthen the ability to notice internal experiences with less automatic reaction.

Buddhist psychology, however, differentiates earlier stages of experience. In the Satipaṭṭhāna framework, *vedanā* refers to the immediate affective tone of experience (pleasant, unpleasant, or neutral) prior to cognitive interpretation. Mental reactions and conceptual narratives arise subsequently. Practice therefore targets reactivity at the level of feeling tone and the mental states that follow it, rather than primarily at the level of cognitive content.

The models differ when the sequence of experience intervention is directed. Western approaches often work with appraisal, belief, and behavioral response, whereas the Buddhist model explicitly trains awareness of pre-conceptual affective processes and their ethical qualities.

#### The potential benefits of contemplating the mind foundation

Incorporating a two-stage process into MBIs could create multiple points for mindfulness to intervene. The first stage involves awareness of raw sensory experience prior to cognitive bias, allowing interruption of automatic emotional reactions. The second stage operates at the conceptual or narrative level, enabling recognition of the mental states that generate cognitive elaboration and habitual patterns. Distinguishing these stages may support intervention at both sensory and cognitive levels, reducing reactivity and promoting more adaptive mental states. This approach parallels MiCBT’s emphasis on interoceptive awareness but extends it by applying mindfulness more explicitly to mental states. As Anālayo (2003, p. 189) notes, “the task of mindfulness is to remain receptively aware by clearly recognizing the state of mind that underlies a particular train of thoughts or reactions.” This is an area for future research.

A recent study by Nicolardi et al. (2024) has begun exploring the relationship between pain and Buddhist mental processes, introducing the concept of the “two arrows of pain.” This model suggests that suffering arises first from direct physical pain and second from reactive mental processes such as aversion and self-identification. Their findings suggest that meditation may modulate the interaction between sensory input and mental factors in pain perception. Further research could examine whether this model applies to other emotional pain, such as the distress experienced in depression or anxiety, and how MBIs might mitigate these effects.

Building on this, traditional Buddhist frameworks provide specific antidotes for unwholesome mental factors (Feldmann, 2016; Gyel-tsen, 2003), raising the possibility that integrating antidote-based strategies into MBIs could enhance their effectiveness. For example, when a practitioner recognizes *anger*, they could be guided to cultivate *non-hatred* and patience as antidotes. This could be incorporated into mindfulness practices, for example, where individuals observe their emotional states nonjudgementally, identify the associated afflictive mental factors, and then engage in intentional practices to break habitual reactive patterns. For example, loving-kindness meditation could be used for *anger* or gratitude exercises for *attachment*.

### Mindfulness of Dhamma

The Dhamma foundation of the Satipaṭṭhāna Sutta is the most philosophically complex of the four foundations and is closely tied to core Buddhist teachings. Similarity ratings indicated that it was the lowest-rated foundation in MBCT (equal with the mind foundation) and the second lowest in MiCBT, both of which are designed for secular clinical audiences. The Dhamma foundation addresses principles such as the cessation of suffering, impermanence, and non-self within a broader soteriological framework. While MBIs also aim to reduce suffering, they do so within a clinical framework focused on emotional regulation and psychological functioning rather than engagement with the metaphysical or liberation-oriented context of traditional Buddhism.

At the same time, MBIs explicitly cultivate reduced reactivity to thoughts and emotions, increased acceptance, and greater behavioral flexibility. In this sense, they promote a form of psychological insight grounded in observing internal experience and disengaging from automatic cognitive patterns. The distinction drawn in the present analysis therefore concerns the scope and framing of this insight when compared with the broader ontological and ethical aims articulated in the Satipaṭṭhāna Sutta.

In traditional Buddhist practice, the first two foundations function as preparatory practices supporting insight into impermanence, non-self, and the Four Noble Truths. From this perspective, the fourth foundation integrates the others into a comprehensive framework for understanding and overcoming suffering. The comparatively limited representation of this foundation in MBCT and, to a lesser extent, MiCBT reflects a divergence in philosophical scope and soteriological framing, rather than a difference in therapeutic intent.

This divergence raises important questions about how much of the original philosophical framework can or should be preserved when adapting Buddhist practices for secular use. Noted Buddhist scholar Thupten Jinpa cautions against teaching complex Buddhist philosophy to those without a foundational understanding of its interconnected philosophies, as this can lead to misunderstandings or misrepresentation of the Buddha’s teachings (Shonin and Van Gordon, 2016), particularly for ideas such as emptiness or non-self. However, there may be secular aspects of Buddhist philosophy that could be adapted for practical use. His Holiness the Dalai Lama has collaborated with Western scientists to integrate aspects of Buddhist and Western thought (Irawan, 2018). Many Western Buddhist scholars, such as Steven Heine, Alan Wallace, Jeffrey Hopkins and Robert Thurman, have successfully found ways to bridge Buddhist philosophy with Western audiences (Heine and Prebish, 2003; Palitsky et al., 2023). Together, these developments suggest that contemporary research is already supporting interventions that retain philosophical depth while remaining accessible.

Recent efforts to bridge this gap include interventions such as Meditation Awareness Training (MAT; Van Gordon et al., 2014) and Mindfulness-Based Intervention incorporating Concentration-, Ethics-, and Wisdom-based practices (MBI-CEW; Furnell et al., 2024). These programs explicitly integrate Buddhist-derived elements of Dhamma, including ethical reflection, insight into impermanence and interconnectedness, and structured development of concentration. Such models demonstrate potential in addressing limitations of first-wave MBIs like MBCT, which have been criticized for omitting wisdom and aspects from the last two foundations of mindfulness, such as the mental factors and the Four Noble Truths (Anālayo, 2019; Monteiro, 2020). These omissions may reduce the transformative potential of mindfulness and risk reinforcing a narrow, individualized focus.

The exclusion of these wisdom-oriented components can have important implications. Without mindfulness of mind and dhamma, MBIs may overlook core insights central to Buddhist practice, such as the impermanent and conditioned nature of mental states, and the ethical dimension of experience. This could limit the mindfulness’s capacity to support deeper self-understanding and long-term behavioral change. Monteiro et al. (2015) warn that this may contribute to *ontological addiction*, where one can unintentionally become more attached to one’s sense of self. While contemporary MBIs do emphasize loosening identification with thoughts at a psychological level, the absence of mindfulness of mind and dhamma may limit engagement with deeper Buddhist insights into the impermanent and conditioned nature of self and identity. In this context, mindfulness may risk being oriented toward improving or managing the self, rather than undermining reification of selfhood altogether. Additionally, when mindfulness is framed primarily as a symptom-reduction tool, rather than a path to insight and compassion, it may inadvertently promote forms of individualism inconsistent with the interdependent view central to Buddhist psychology. A more holistic integration of all four foundations could offer a richer and more sustainable framework for wellbeing and mental health and is an area for future research.

To better balance these limitations, further integrating ethical, spiritual and wisdom-based frameworks into MBIs could enrich their scope and long-term impact. Such a shift not only addresses concerns about philosophical dilution but also supports deeper reflection on whether one’s mental states and behaviours contribute to both personal and collective well-being. Rather than treating all thoughts and emotions as neutral, individuals could apply a secular, non-religious, or Buddhist moral framework, similar to how Acceptance and Commitment Therapy (ACT) incorporates values (Hayes and Pierson, 2005). While ACT promotes a non-judgmental, values-driven approach, Buddhist psychology emphasizes the distinction between wholesome and unwholesome intentions, offering more specific guidance. Integrating such frameworks into MBIs may foster long-term behavioral change by aligning actions with values that promote wellbeing beyond symptom reduction (Perera et al., 2025). Future research could further explore which elements of Buddhist ethics and wisdom could be adapted into clinical settings without compromising their philosophical depth.

### Limitations

This work has several limitations. First, the scope was intentionally constrained given the diversity of Buddhist traditions and their Western adaptations. Relying on a single text to represent Buddhist mindfulness limits comprehensiveness, as the Buddha offered multiple teachings relevant to mindfulness. While the Ānāpānasati Sutta is another foundational text integrating the four foundations into a breath-based practice (Anālayo, 2019), the Satipaṭṭhāna Sutta was selected for its clearer structural delineation of body, feelings, mind, and dhammas. Given that MBIs emphasize generalized awareness rather than breath-specific techniques, the broader scope of the Satipaṭṭhāna Sutta provided a more suitable benchmark for cross-program comparison.

Second, the analysis was limited to two clinically oriented MBIs, enabling in-depth comparison but necessarily narrowing the interpretive scope. This focus contributed to the finding that mindfulness of mind and dhammas were less integrated than the first two foundations; however, it remains unclear whether non-clinical or educational MBIs might show broader alignment. Even so, the template analysis provides a model for future comparisons.

Notably, although MBSR occupies a foundational place in the development of MBIs and was originally developed for stress in medical populations, it has since been applied across a wide range of clinical contexts. It was not included in the present analysis because the study required interventions with psychological disorder-specific treatment protocols and manuals, allowing for a more focused comparison. Its exclusion therefore reflects the analytic scope of the study rather than any assessment of MBSR’s clinical breadth or effectiveness. Additionally, previous analyses suggest limited alignment between MBSR and the four foundations (Anālayo, 2019).

Thirdly, MBIs are not designed to be Buddhist practices, and thus, their alignment with traditional Buddhist concepts, such as liberation, is not inherently a goal. Participants typically engage with MBIs for psychological benefits rather than spiritual goals, and their engagement with mindfulness practices may differ from the spiritual goals emphasized in Buddhism.

Fourth, manual-based analyses omit the nuances of oral transmission. MBCT and MiCBT instructors often convey meaning through experiential, relational, or implicit teaching, which cannot be fully captured in written materials. Future research could combine manual analysis with session recordings or trainer interviews to better reflect how mindfulness is embodied in practice.

Finally, this analysis reflects the perspective of a single author with a deeper understanding of Buddhism then MBI’s, which may have introduced biases regarding interpretations. While this limitation was addressed through the methodology and validation protocols, incorporating a broader range of perspectives during the construction of the template could have further mitigated this issue.

## Conclusion

This study identified a fundamental divergence between the Satipaṭṭhāna Sutta and contemporary mindfulness-based interventions. Whereas the Satipaṭṭhāna Sutta presents a comprehensive framework encompassing mindfulness of body, feelings, mind, and dhammas - embedded within ethical and ontological aims - MBCT and MiCBT operationalize mindfulness within psychological and clinical frameworks oriented toward symptom reduction and emotional regulation.

Clear differences also emerged between MBCT and MiCBT. Although both align most strongly with the Sutta’s first two foundations, their alignment decreases as the text moves into more cognitive, ethical, and philosophical territory. This divergence is more pronounced in MBCT, which takes a broader, more eclectic clinical approach, whereas MiCBT remains more structurally aligned with Buddhist frameworks and incorporates elements more directly derived from the four foundations. As a result, MiCBT more closely reflects the Sutta’s traditional intent, while MBCT represents a more secularized and psychologically focused adaptation.

The findings indicate scope for integrating selected elements of Buddhist psychological theory into MBIs. These include clearer differentiation of feeling tone, incorporation of models of cognition involving mental states and mental factors, and limited engagement with ethical reflection, which may be particularly relevant in an increasingly complex and changing social context (King, 2009). Such developments may enhance the conceptual coherence and long-term effects of mindfulness-based interventions without requiring religious framing.

## Data Availability

The data supporting the conclusions of this article are available from the corresponding author on reasonable request.
