# Supplementary material for: Bridging traditions: a template analysis of Buddhist and Western psychological approaches to mindfulness
Source: Front Psychol. 2026 Jun 10;17:1711922. doi: 10.3389/fpsyg.2026.1711922 (PMC13290574; doi:10.3389/fpsyg.2026.1711922)
Supplement: Supplementary file 1 [file Table_1.docx]

**Supplementary Table S1**
*Exemplar passages from the Sattipatthana Sutta and corresponding MBCT and MiCBT manual illustrating coding decisions for similarity ratings.*

| **Foundation of Mindfulness** | **Satipaṭṭhāna Sutta Passage** | **MBCT Excerpt / Session** | **MiCBT Excerpt / Session** | **Alignment Summary and Foundation Rating** |
| --- | --- | --- | --- | --- |
| Body   Subcategory: breathing | “Ever mindful he breathes in; mindful he breathes out” | Session 3 – Breath and Body Awareness Practice: Guides participants to attend to the natural breath and body sensations without control, anchoring attention in the present.  (Rating = 6) | Session 2 – Mindfulness of Breath: Participants observe sensations throughout the body and develop equanimity toward arising discomfort.  (Rating = 6) | Both programs closely align with the Sutta’s instructions on mindfulness of breathing, using awareness of the natural breath and embodied sensations as the primary anchor for cultivating sustained attention, recognizing distraction, and returning awareness to the body in the present moment. |
| Feelings  Subcategory:  Affective qualities | “He knows a pleasant feeling as pleasant, an unpleasant feeling as unpleasant, a neutral feeling as neutral.” | \| Session 4 - Introduces recognising experiences as pleasant, unpleasant or neutral, but the primary therapeutic focus is on negative affect and aversion linked to depression and rumination. Pleasant/neutral categories are acknowledged only to the extent they inform relapse-prevention strategies (e.g., Pleasant Events Calendar).  (Rating = 4) \| \| --- \| | Session 3 & 4 – Equanimity Training: Body scanning to observe pleasant and unpleasant sensations without reactivity, explicitly linking awareness of bodily feeling tone to equanimity.  Rating = 5) | While both MBCT and MiCBT recognize pleasant, unpleasant, and neutral affective tones, the Sutta situates this awareness within the broader cultivation of non-attachment and equanimity. MBCT recognizes affective tone categories but operationalizes them psychologically toward mitigating negative mood rather than phenomenologically exploring all tones. MiCBT’s focus on non-reactivity to bodily sensation aligns more closely with the Sutta’s equanimity and vedanā as a universal mechanism in the arising and cessation of suffering |
| Mind  Subcategory:  Ordinary States | “Herein, monks, a monk knows the consciousness with hate, as with hate; the consciousness without hate, as without hate;’” | Session 7 & 8 – MBCT encourages recognizing thoughts and emotions as passing mental events and responding with non-judgmental awareness to reduce rumination and depressive relapse. However, MBCT does not classify mental states as helpful or harmful and does not include ethical or spiritual appraisal, which resulted in a lower similarity rating.  (Rating = 3) | Session 9 - Notes that “doing harm to oneself or to others is likely to increase distress caused by the act itself, by subsequent guilt and self‐blame, and by the possible interpersonal conflicts caused by the action” (Cayoun et al., 2019, p. 287), reflecting a practical ethical appraisal of mind states that is more consistent with the Sutta’s distinction between ordinary and wholesome mental qualities.  (Rating = 4) | Both MBCT and MiCBT do not explicitly integrate the mind foundation’s distinction of ordinary states of mind, but they do emphasize recognizing the mind’s current state and whether certain mental patterns are helpful or unhelpful. However, MiCBT received a slightly higher similarity rating because, unlike MBCT’s focus on noticing mental patterns without an ethical or spiritual frame, MiCBT explicitly distinguishes between helpful and hindering states and incorporates practical ethical guidance, which reflects a closer conceptual correspondence with how mental states are evaluated in the Sutta. |
| Dhamma  Subcategory:  The Four Noble Truths | “Herein, monks, a monk knows, *"This is suffering,"* according to reality; he knows, *"This is the origin of suffering,"* according to reality; he knows, *"This is the cessation of suffering,"* according to reality; he knows *"This is the road leading to the cessation of suffering,"* according to reality.” | Session 5 - MBCT supports recognizing the nature of suffering by helping individuals notice how depressive thinking contributes to distress, particularly in Session 5’s focus on rumination and negative thinking patterns. However, MBCT does not explicitly reference the Four Noble Truths or the broader spiritual and ethical context in which they are traditionally taught, instead presenting the reduction of suffering within a secular, psychological framework.  (Rating = 2) | Session 7 & 9 - MiCBT makes implicit links to the Four Noble Truths, such as in Session 7’s anecdote explaining why distress arises and in Session 9’s discussion of suffering in relation to ego and compassion. It also partially aligns with the Fourth Noble Truth through behavioral and ethical guidance, including discussion of harmful speech and actions.  (Rating = 4) | Both programs aim to reduce suffering, but MBCT applies this only psychologically, whereas MiCBT incorporates explicit discussions about the causes of suffering and introduces elements related to ethical conduct that partially mirror the Four Noble Truths. |

Note. These excerpts are illustrative rather than exhaustive and reflect the four foundations included in the coding.

1. **Not at all similar (0%)**: The texts share no common themes, content, or meaning.
2. **Little similarity (15%)**: The texts have minimal overlap, with only a few shared ideas or concepts.
3. **Somewhat similar (25%)**: The texts share some common themes but differ significantly in details and presentation.
4. **Moderately similar (50%)**: The texts have an equal balance of similarities and differences, sharing several key themes and ideas with some differences in details or emphasis.
5. **Mostly similar (75%)**: The texts are alike in most aspects, sharing major themes and content, with minor differences.
6. **Very similar (85%)**: The texts are highly alike, with only slight differences in wording or emphasis.
7. **Completely similar (100%)**: The texts are nearly identical in meaning, content, and presentation, with no significant differences
